# Targeted genetic manipulations of neuronal subtypes using promoter-specific combinatorial AAVs in wild-type animals

**DOI:** 10.3389/fnbeh.2015.00152

**Published:** 2015-07-02

**Authors:** Heinrich S. Gompf, Evgeny A. Budygin, Patrick M. Fuller, Caroline E. Bass

**Affiliations:** ^1^Department of Neurology, Division of Sleep Medicine, Harvard Medical School and Beth Israel Deaconess Medical CenterBoston, MA, USA; ^2^Department of Neurobiology and Anatomy, Wake Forest School of MedicineWinston Salem, NC, USA; ^3^Department of Biology, St. Petersburg State UniversitySt. Petersburg, Russia; ^4^Department of Pharmacology and Toxicology, School of Medicine and Biomedical Sciences, University at BuffaloBuffalo, NY, USA

**Keywords:** dual AAV targeting, wild-type, optogenetic, chemogenetic, DREADD, tyrosine hydroxylase (TH), ventral tegmental area (VTA), locus coeruleus (LC)

## Abstract

Techniques to genetically manipulate the activity of defined neuronal subpopulations have been useful in elucidating function, however applicability to translational research beyond transgenic mice is limited. Subtype targeted transgene expression can be achieved using specific promoters, but often currently available promoters are either too large to package into many vectors, in particular adeno-associated virus (AAV), or do not drive expression at levels sufficient to alter behavior. To permit neuron subtype specific gene expression in wildtype animals, we developed a combinatorial AAV targeting system that drives, in combination, subtype specific Cre-recombinase expression with a strong but non-specific Cre-conditional transgene. Using this system we demonstrate that the tyrosine hydroxylase promoter (TH-Cre-AAV) restricted expression of channelrhodopsin-2 (EF1α-DIO-ChR2-EYFP-AAV) to the rat ventral tegmental area (VTA), or an activating DREADD (hSyn-DIO-hM3Dq-mCherry-AAV) to  the  rat  locus  coeruleus  (LC). High expression levels were achieved in both regions. Immunohistochemistry (IHC) showed the majority of ChR2+ neurons (>93%) colocalized with TH in the VTA, and optical stimulation evoked striatal dopamine release. Activation of TH neurons in the LC produced sustained EEG and behavioral arousal. TH-specific hM3Dq expression in the LC was further compared with: (1) a Cre construct driven by a strong but non-specific promoter (non-targeting); and (2) a retrogradely-transported WGA-Cre delivery mechanism (targeting a specific projection). IHC revealed that the area of c-fos activation after CNO treatment in the LC and peri-LC neurons appeared proportional to the resulting increase in wakefulness (non-targeted > targeted > ACC to LC projection restricted). Our dual AAV targeting system effectively overcomes the large size and weak activity barrier prevalent with many subtype specific promoters by functionally separating subtype specificity from promoter strength.

## Introduction

Despite their exceptional value as experimental model systems, rats and other wild-type species have one substantial drawback: their genetic tractability as a model organism falls far short of the mouse. To overcome this, viral vectors have been used in rats to manipulate CNS function (e.g., through delivery of genes, antisense or RNAi) in restricted brain regions of interest. However, viruses indiscriminately transduce most cell types within the region they are introduced. Since the brain is highly heterogeneous, there is a clear need to manipulate neuronal subtypes independently in order to: (1) understand the genetic basis of neurobiological phenomena; and (2) selectively target regions and cell groups for therapeutic purposes. Rat Cre driver lines have been developed and will provide a similar level of selectivity to mouse models, but they are not yet common and like transgenic mice they require a significant investment in time and resources to maintain. Thus there remains a clear need to pursue other techniques.

Adeno-associated virus (AAV) has proven to be very effective for studying neuronal function and behavior. It is long-lived, highly neurotropic, non-pathogenic and, importantly, it can drive transgene expression at significantly high levels necessary to alter neuronal function and produce a behavioral phenotype (McCown, 2011). However, AAV is relatively small with a limited packaging capability, often cited as 4.7–5.4 kilobases (Grieger and Samulski, 2005). Thus AAV cannot be used in targeting strategies that require large cell type specific promoters. In addition, cell type specific promoters are usually relatively weak and unable to drive sufficient levels of expression for experimental purposes. While a few minimal subtype specific promoters are available, their use has been limited to just a very few neuronal subtypes and discrete brain regions (Konadhode et al., 2013; Vazey and Aston-Jones, 2014). Alternatively, combinatorial strategies have been designed to express circuit-manipulating constructs in adult brains by delivering recombinase-dependent viruses into discrete brain regions of recombinase-expressing transgenic animals (Tolu et al., 2010; Fenno et al., 2014). Again, this approach depends on the availability of specific Cre driver lines, of which there are few for rats. Importantly, many physiological phenomena and sophisticated behaviors in rats are strain dependent.

**Figure 1:**
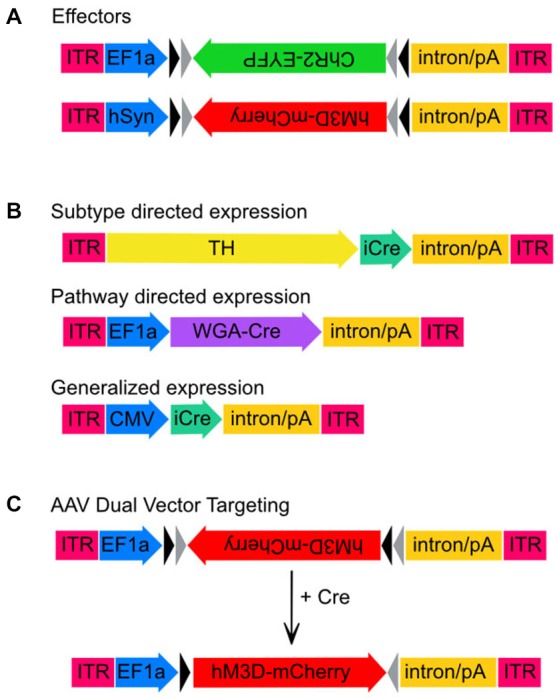
**Adeno-associated virus (AAV) transgenes used in this study.** Schematic maps of AAV vector constructs. **(A)** Effector gene constructs, ChR2 and hM3Dq and the promoters used to drive high neuronal expression rates, EF1-α and hSyn, respectively. Note that both effector molecule sequences are in the DIO position. **(B)** Maps of the three vector constructs used to drive expression of Cre-recombinase. Sizes are approximate and demonstrate that the TH promoter is much larger than either EF1-α or CMV. **(C)** Unlocking of the hM3Dq construct from the DIO orientation to the sense orientation following Cre-mediated recombination.

**Figure 2:**
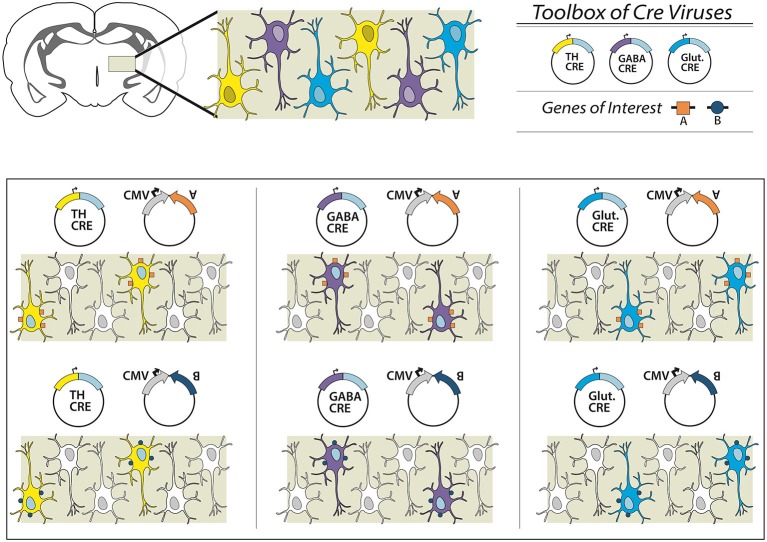
**Overview of the dual virus targeting system for targeted expression of transgenes to specific neuronal subtypes.** In this schematic we present a brain region with three different neuronal subpopulations: TH-expressing (yellow), GABAergic (purple) and glutamatergic (blue) neurons. To examine all three subpopulations individually a researcher could obtain or create an effector (e.g., gene A, excitatory DREADD or ChR2), and combine with a virus from an existing toolbox of neuron subtype-specific promoter-Cre viruses. Depending on the Cre virus chosen, a different neuronal population is targeted. In other experiments, the effector itself can be switched (e.g., gene B), and thus multiple genetic manipulations can be conducted within one specific targeted neuronal subpopulation. In this way, the researcher is only required to create the appropriate DIO-effector construct once, instead of attempting multiple promoter-effector combinations, which may be of limited value given variegations in promoter-gene interaction and weak promoter activity.

Here we present a new combinatorial dual AAV vector approach to achieve specificity while maintaining high expression levels in wildtype animals, namely rats. The first AAV incorporates the gene of interest in a double-inverted open (DIO) reading frame, (Figure 1A) driven by a strong neuron specific or generalized promoter (human synapsin, hSyn or elongation factor-1 alpha, EF1α, respectively). In the absence of Cre the gene is retained in an inverse, non-sense orientation. The second AAV delivers Cre-recombinase under control of a subtype specific promoter (1b). We hypothesize that only small amounts of Cre are needed to reorient the transgene for expression, thus a significantly weaker, but neuronal subtype-specific promoter—in this case tyrosine hydroxylase (Oh et al., 2009)—can drive sufficient Cre expression. In combination, these AAVs enable expression of a Cre-dependent transgene in a cell-type (TH+) specific pattern (1c). This approach then allows the flexibility of using viral vector constructs expressing different effectors (e.g., excitatory or inhibitory channelrhodopsins, DREADDS and other genes of interest) with the same promoter-Cre AAV (e.g., TH-Cre-AAV), or limiting a transgene to different neuronal subpopulations utilizing a toolbox of verified promoter-Cre constructs. For example, a single EF1α-DIO-gene of interest can be combined with a Cre virus driven by promoters that restrict to glutamatergic neurons in the targeted brain region (e.g., the CamK2α promoter in the hippocampus, forebrain or amygdala; Minichiello et al., 1999). Alternatively, GABAergic neurons may possibly be targeted with a vesicular GABA transporter (VGAT) or GAD67 promoter (Figure 2).

To validate this system *in vivo* we have used: (1) a Cre-conditional channelrhodopsin-2 (ChR2), co-infused with a TH-Cre-AAV to restrict expression of ChR2 to TH+ neurons of the ventral tegmental area (VTA); and (2) a Cre-conditional excitatory DREADD, co-infused with a TH-Cre-AAV to restrict expression of the hM3Dq to TH+ neurons of the locus coeruleus (LC). We compared the effectiveness of TH-targeted Cre with a Cre-recombinase driven by a non-selective, strong cytomegalovirus promoter (CMV-Cre-AAV) and a Cre-recombinase targeted to the LC via retrograde transport (Gradinaru et al., 2010; WGA-Cre-AAV) from a LC target area, the anterior cingulate cortex (ACC). Together our results demonstrate that dual AAV targeting of TH+ neurons in the VTA and LC in wild-type animals, likely reflecting dopaminergic or noradrenergic neurons, is effective and the amount of recruited TH+ neurons appears proportional to the behavioral effects.

## Materials and Methods

### Animals

Adult male Sprague Dawley rats (275–300 g; Harlan, Indianapolis, IN, USA) were individually housed and had free access to food and water. All animals were housed under controlled conditions (12 h light/dark cycle, starting at 7:00 A.M.; 100 lux) in an isolated ventilated chamber maintained at 20–22°C. All protocols were approved by the Institutional Animal Care and Use Committees of Beth Israel Deaconess Medical Center, Harvard Medical School, Wake Forest School of Medicine and the University at Buffalo.

### Genetic Constructs

We initially tested the dual AAV targeting system with a channelrhodopsin-2 (ChR2) construct for light induced neuronal firing. This construct expressed ChR2 under the control of the elongation factor 1 alpha promoter (EF1α-DIO-ChR2-EYFP-AAV, hereafter ChR2-AAV, a kind gift from K. Deisseroth), and also included an enhanced yellow fluorescent protein (EYFP) fusion, which aids in immunohistochemical determination of ChR2 expression. In addition, we used the excitatory M3 DREADD (“Designer Receptors Exclusively Activated by Designer Drugs”) packaged into the hSyn-DIO-hM3Dq-mCherry-AAV (hM3Dq-AAV, a kind gift from B. Roth) virus to test our combinatorial viral vector approach. Bryan Roth and colleagues used a directed genetic evolution technique to develop muscarinic receptors that are insensitive to acetylcholine but highly responsive to the clozapine metabolite clozapine-N-oxide (CNO; Armbruster et al., 2007; Nawaratne et al., 2008). These DREADDs have now been used in various neuronal populations to control neuronal activity non-invasively (Alexander et al., 2009; Ferguson et al., 2011). The transgenes in both of these constructs were packaged in a double-inverted open (DIO) reading frame (Figure 1).

To obtain neuronal subtype specificity for TH neurons we used a 2.5 kb segment of the rat TH promoter that has previously been shown to drive expression predominantly in TH neurons including in the LC (Oh et al., 2009). The 2.5 kb TH promoter was cloned into the pAAV vector to create TH-pAAV. The codon optimized Cre recombinase gene (improved Cre or iCRE) was subsequently inserted 3′ of the TH promoter (TH-Cre-AAV). Another construct used to compare the subtype-specific expression of TH-Cre-AAV with non-subtype specific expression was a virus constitutively expressing Cre under the control of the CMV promoter (referred to here as CMV-Cre-AAV to distinguish it from the TH-Cre-AAV construct). Finally, to achieve trans-synaptic transport of Cre-recombinase we used a recently described wheat-germ agglutinin (WGA)-Cre fusion protein (Gradinaru et al., 2010) in which the WGA portion of the fusion protein facilitates retrograde transport of Cre to the cell bodies of anatomically connected neurons. This fusion protein was expressed under the control of the EF1α promoter (WGA-Cre-AAV).

### AAV Packaging

All constructs were packaged using the standard triple transfection protocol in HEK-293 cells to create recombinant pseudotyped AAV2/10 virus (Xiao et al., 1998). AAV2/10 virus is highly neurotrophic, exhibits enhanced spreading compared to other commonly used serotypes, and has previously been used by our laboratories to drive high levels of expression in both extremely small and large brain regions (Lazarus et al., 2007; Bass et al., 2013; Anaclet et al., 2014; Fenno et al., 2014). Viruses were titered using quantitative PCR and were approximately 10^12^ vector genome copies per ml.

### Study Design

Rats received intracranial injections of different AAVs and in varying combinations that included: Experiment (1) TH-Cre-AAV co-injected with ChR2-AAV in the VTA; (2) TH-Cre-AAV co-injected with the hM3Dq-AAV in the LC; (3) AAV expressing Cre under the strong constitutively active CMV promoter (CMV-Cre-AAV), co-injected with hM3Dq-AAV in the LC; and (4) WGA-Cre-AAV injected into the ACC and hM3Dq-AAV into the LC.

### Surgery

For optogenetics experiments, rats were anesthetized with ketamine hydrochloride (100 mg/kg, i.p.) and xylazine hydrochloride (20 mg/kg, i.p.), infused with virus and implanted with a fiber optic cannula. Two small holes were drilled and two skull screws were placed in to secure a cement cap. A third hole was drilled above the right VTA (5.8 AP; 0.7 ML) and an optic-fluid cannula (OFC; Doric Lenses, Canada) was implanted (7.3 DV). A total of 240 nl of TH-Cre-AAV + 960 nl ChR2-AAV were infused over 3 min. The exposed skull was coated with dental cement secured by skull screws. For DREADD experiments, under chloral hydrate anesthesia (7% in saline; 350 mg/kg), fine glass pipettes for the intracranial injections of different AAVs were lowered to a pre-calculated target bilaterally into the anterior cingulate cortex (ACC; coordinates: 1.25 AP, ±0.5 ML, 1.0 DV) or bi-laterally into the LC (coordinates: −9.7 AP, ±1.3 ML, 6.4 DV) based on the rat atlas of Paxinos and Watson (Watson, 1998) and the solution (600 nl of a mix of equal parts Cre-AAV and hM3Dq-AAV LC bilateral; 900 nl Wga-Cre-AAV ACC bilateral, 300 nl hM3Dq-AAV LC bilateral; 600 nl of a mix of equal parts TH-Cre-AAV and hM3Dq-AAV LC bilateral) was injected by an air pressure system.

### Optical Delivery and Fast-Scan Cyclic Voltammetry

For the evaluation of changes in extracellular dopamine concentrations in response to optical stimulation of dopamine cell bodies, rats (*n* = 5) were anesthetized with urethane (1.5 g/kg, i.p.) and placed in a stereotaxic frame. The cement cap was removed and a hole for a carbon fiber electrode (~100 μm in length, 6 μm in diameter) insertion was drilled (from bregma: anterior-posterior, 1.2 mm; lateral, 2 mm). An Ag/AgCl reference electrode was implanted in the contralateral hemisphere and a carbon fiber electrode was positioned in the nucleus accumbens core (dorsal-ventral, 7.2 mm). The optical fiber (200 μm in diameter), used for optical activation of ChR2 expressing cells, was positioned at the level of the dopamine cell body region 300 μm above the virus injection point (unilateral). The reference and carbon fiber electrodes were connected to a voltammetric amplifier (UNC Electronics Design Facility, Chapel Hill, NC, USA) and voltammetric recordings were made at the carbon fiber electrode every 100 ms by applying a triangular waveform (−0.4 to +1.3 V, 300 V/s). Light evoked dopamine release was identified by the background-subtracted cyclic voltammogram. Carbon fiber microelectrodes were calibrated *in vitro* with known concentrations of dopamine (2–5 μM). To analyze dopamine release and uptake parameters, light-evoked changes in extracellular dopamine were fit using the equation:

$$\text{d}\left[\text{DA}\right]/\text{dt}\text{ = }(\left(f\right){\left[\text{DA}\right]}_{\text{p}}-\left(V_{\text{max}}/\left(\left\{K_{\text{m}}\text{/}[\text{DA}\right]\right)\text{ + 1}\right)$$

where *f* is the stimulation frequency (Hz), [DA]_p_ is the concentration of dopamine released per light pulse, and *V*_max_ and *K*_m_ are Michaelis-Menten rate constants for dopamine uptake (Garris and Rebec, 2002; Phillips et al., 2003; Oleson et al., 2009; Pattison et al., 2011).

The optical stimulation setup was reported in previous work (Bass et al., 2010) and consisted of a laser at wavelength 473 nm (Beijing Viasho Technology Co., Ltd., Beijing, China) with a maximum output of 100 mW. The laser was modulated using the TTL input control port on the laser power supply via a USB Data Acquisition unit (National Instruments 6221-USB). The data acquisition unit was controlled by a desktop computer using LabVIEW software (National Instruments). The software allowed us to control the frequency of the square pulses, the total number of pulses in one data stream, and the width of each pulse. The laser power output was measured using a commercial power meter (Newport Model 1815C).

### EEG/EMG Recording and Sleep Analysis for DREADD Experiments

After animals were anesthetized with chloral hydrate and injections were performed as above, the skulls were exposed. Four EEG screw electrodes were implanted into the frontal (two) and parietal bones (two) of each side of the skull, and two flexible EMG wire electrodes were placed into the neck muscles. The free ends of the leads were fitted into a socket that was attached to the skull with dental cement. At least 2 weeks after surgery, the sockets were connected via flexible recording cables and a commutator to an amplifier (A-M Systems model 3600, Carlsborg, WA, USA) and computer, and signals were digitized using a Dell PC running the Sleep Sign recording system (Kissei Comtec, Irvine, CA, USA). The EEG/EMG was recorded at the end of the second week after surgery, for 48 h. Injections of CNO (0.3 mg/kg) or saline were performed at ZT 5, 24 h into this continuous recording period in a cross-over design (animals received either saline or CNO first), and injections were at least 1 week apart from one another to allow sufficient time for CNO washout and recovery. The cages were placed in such a way that animals receiving the same treatment were located next to one another so that the CNO-injected rat did not keep the saline-injected rat awake. The only exception to this were the sham virus, CNO injected animals in Figure 7D.

Wake-sleep states were automatically scored and manually checked in 4 s epochs on the digitized EEG/EMG. Wakefulness was identified by the presence of a desynchronized EEG and phasic EMG activity. Additionally, wakefulness was distinguished from sleep states by use of video monitoring. Nonrapid eye movement (NREM) sleep consisted of a high amplitude slow wave EEG together with a low EMG tone relative to wake. REM sleep was identified by the presence of regular theta EEG activity coupled with low EMG tone relative to NREM sleep. The amount of time spent in wake, NREM sleep, and REM sleep was determined for each 60 min period and treatment groups were compared using the student’s *t*-test performed in Microsoft Excel.

### Immunohistochemistry (IHC)

Animals were deeply anesthetized with isoflurane and then perfused with 50 ml saline followed by 500 ml of 10% formalin through the heart. The brains were removed, postfixed for 2 h in 10% formalin, and then equilibrated in 20% sucrose in PBS containing azide overnight. The brains were sectioned on a freezing microtome at 40 μm into four series. Sections were washed in 0.1 M PBS, pH 7.4 (two changes), and then incubated in the primary antiserum for 1 day at room temperature. For c-Fos, we used a rabbit polyclonal antiserum (AB5; 1:50,000; Oncogene Sciences, Cambridge, MA, USA) against residues 4–17 from human c-Fos. This antiserum stains only the nuclei of neurons based on recent activity patterns (Gaus et al., 2002; Lu et al., 2002). For Cre, we used a rabbit polyclonal antiserum (Novagen, lot #D00132036, catalog #69050, 1:10,000). Sections were then washed in PBS and incubated in biotinylated secondary antiserum (1:1000 in PBS) for 1 h, washed in PBS, and incubated in avidin-biotin-horseradish peroxidase conjugate (Vector Laboratories) for 1 h. Sections were then washed again and incubated in a 0.06% solution of 3, 3-diaminobenzidine tetrahydrochloride (DAB; Sigma, St. Louis, MO, USA) plus 0.02% H_2_O_2_. The sections were stained brown with DAB only or black by adding 0.05% cobalt chloride and 0.01% nickel ammonium sulfate to the DAB solution. For double immunofluorescence, sections were rinsed in PBS for 5 min and again in PBS + 0.5% triton X-100 three times for 10 min. Sections were incubated with primary antibodies overnight at 4°C. Primary antibodies consisted of a mouse anti-tyrosine hydroxylase (ImmunoStar #22941, 1:4000) and a rabbit anti-GFP (Invitrogen #A6455, 1:2000) diluted in PBS + 0.3% triton X-100. For single DsRed immunofluorescence, we used a rabbit polyclonal antiserum (632496; 1:10,000; Clontech Laboratories, Inc., Mountain View, CA, USA). The antibody was raised against the full-length DsRed-Express protein and recognizes wt DsRed and its variants (Yang et al., 2013). The next day the sections were rinsed three times for 10 min each in PBS and then incubated with secondary antibodies consisting of Alexa 555 donkey anti-mouse (Invitrogen #A31570, 1:4000) and Alexa 488 goat anti-rabbit (Invitrogen #A11034, 1:2000) for the double immunofluorescence experiments and in red fluorescent Cy3 conjugated donkey anti rabbit (1:10,000; 103422; Jackson ImmunoResearch, West Grove, PA, USA) for 2 h at room temperature. Sections were rinsed again in PBS three times for 10 min, mounted on slides and coverslipped using Prolong Gold media. Slides were visualized on a Zeiss Axio Observer Confocal microscope. For studies examining colocalization of TH+ and ChR2-EYFP+ neurons, we performed immunohistochemistry (IHC) on sections collected from animals injected with the TH-Cre-AAV + ChR2-AAV (*n* = 4). Z-stacks (approximately 35–40, 1.140 μm optical slices) from several fields in the VTA were collected and examined using the Zeiss Zen software. ChR2+ cell bodies were carefully examined in each z-stack to determine if a TH signal was also present.

## Results

### Functional Targeting of ChR2 Receptors to TH Neurons of the VTA

**Figure 3:**
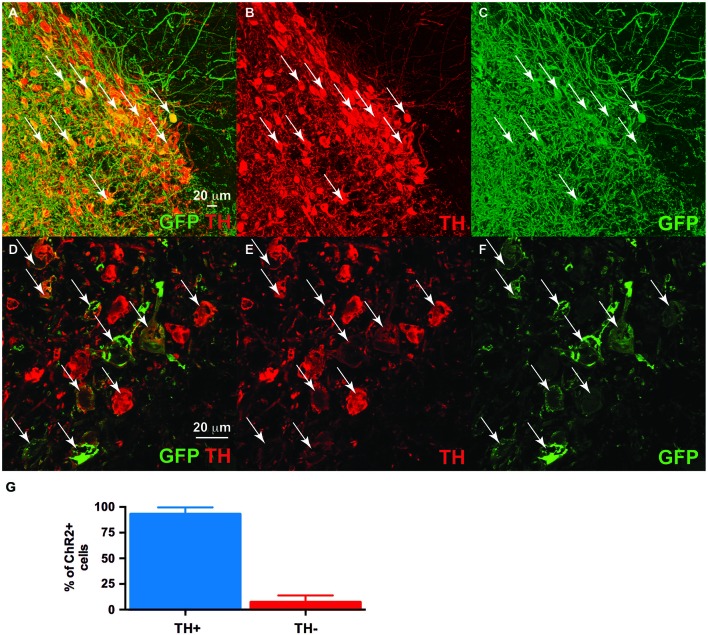
**Dual ventral tegmental area (VTA) injection of TH-Cre-AAV and ChR2-AAV.** Immunofluorescence of **(C,F)** ChR2 expression (green). **(B,E)** TH expression (Red). **(A,D)** Merged image of band **(C)** and **(B)**, and **(E)** and **(F)**, respectively. Note ChR2 expression is limited to TH-expressing neurons, with all ChR2+ neurons also demonstrating TH (arrows), while some TH+ neurons are either not expressing ChR2 or doing so at levels below detection limit of this experiment. **(G)** Quantification of TH/ChR2 colocalization. ChR2+ neurons were identified as either TH+ or TH- (*n* = 4, total of 427 cells).

**Figure 4:**
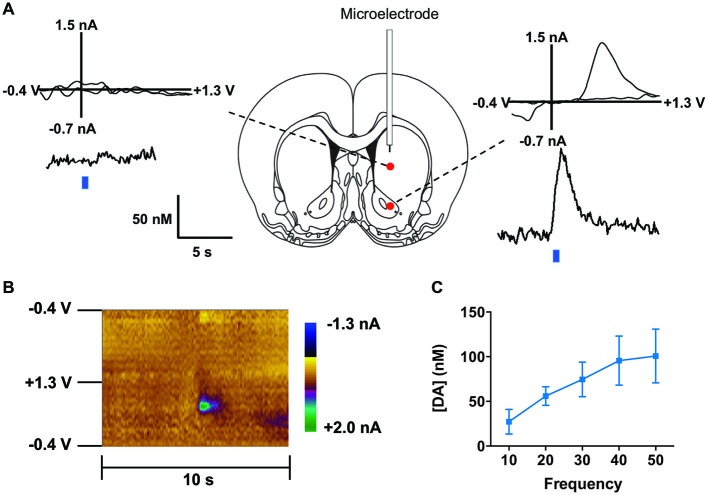
**Optogenetic stimulation of TH+ VTA neurons. (A)** Dopamine changes detected by fast-scan cyclic voltammetry (FSCV) in the striatum of a representative anesthetized rat in response to optical stimulation (50 Hz, 50 pulses) of the middle brain. Top graphs show the background-subtracted voltammogram obtained at the end of the stimulation, which demonstrate the characteristic oxidation and reduction peak potentials (~ +0.6 V and ~ −0.2 V, respectively) that identify dopamine. Bottom traces show the concentration-time plots of optically-evoked dopamine release measured in real time (left from dorsal striatum, right from ventral striatum, blue line indicates time of light pulse). **(B)** Standard two dimensional color plot which topographically depicts the voltammetric data with time on the *x*-axis, applied scan potential on the *y*-axis and background-subtracted faradaic current shown in the *z*-axis in pseudo-color. Dopamine can be seen during stimulation at the signature 0.65 V (oxidation peak encoded as green). **(C)** Magnitude of light evoked dopamine release is frequency dependent.

We co-injected TH-Cre-AAV and ChR2-AAV into the VTA and these animals demonstrated highly specific ChR2 expression (Figures 3A–F) in TH+ neurons. Several sections from different subjects were carefully examined to determine the degree of colocalization between TH and ChR2. A total of 427 ChR2+ cells were counted, of which 399 were determined to also express TH, while 28 did not, resulting in approximately 93.4% of colocalization. In Figure 3G the mean % ChR2+ neurons that were also TH+ or TH- is represented. We then employed *in vivo* fast-scan cyclic voltammetry (FSCV) to determine if the level of ChR2 expression achieved in the VTA is sufficient for optical stimulation of dopamine release in the rat nucleus accumbens. Figures 4A,B demonstrates dopamine efflux, triggered by blue-light stimulation of the VTA (50 Hz, 50 flashes with 4-ms flash length, 3 mW). Notably, dopamine release was restricted to the ventral striatum and largely absent in the dorsal striatum, which receives dopaminergic inputs primarily from the substantia nigra. The rising fraction of accumbal dopamine efflux was time-locked to the 1-s VTA optical stimulation (Figure 4A), whereas dopamine concentration rapidly declined immediately at the end of the stimulation due to the reuptake mechanism. The magnitudes of dopamine uptake parameters, including maximal velocity of dopamine reuptake rate or *V*_max_ and apparent *K*_m_ (Michaelis-Menten constant) were similar to those previously found for the rat nucleus accumbens core using FSCV *in vivo* (Phillips et al., 2003; Jones et al., 2006; Oleson et al., 2009; Pattison et al., 2011, 2012). *V*_max_ values were between 1.74 and 3.37 μM/s, when *K*_m_ was set between 0.16 and 0.2 μM (*n* = 5). The responsiveness of dopamine release to different frequencies (from 10 to 50 Hz, 1 s total duration) also was evaluated. The magnitudes of light-evoked dopamine release were frequency dependent (*F*_(4,6)_ = 9.085, *p* < 0.0001, *n* = 5; one-way ANOVA). The maximal increase in dopamine release was observed when ChR2 expressing dopamine neurons of the VTA were stimulated at the 40 Hz frequency. The averaged values of dopamine release were 27.2 ± 13.8 nM for 10 Hz, 55.9 ± 10.4 nM for 20 Hz, 74.6 ± 19.3 nM for 30 Hz, 95.6 ± 27.4 nM for 40 Hz and 100.8 ± 30.7 nM for 50 Hz stimulation (Figure 4C). This exclusive frequency dependence is likely based on the biological properties of the ChR2 proteins. According to electrophysiological studies, many cells cannot follow ChR2-driven spiking above the 40 Hz range in sustained trains (Lin et al., 2009; Gunaydin et al., 2010).

### Functional Targeting of hM3Dq Receptors in TH Neurons of the LC

**Figure 5:**
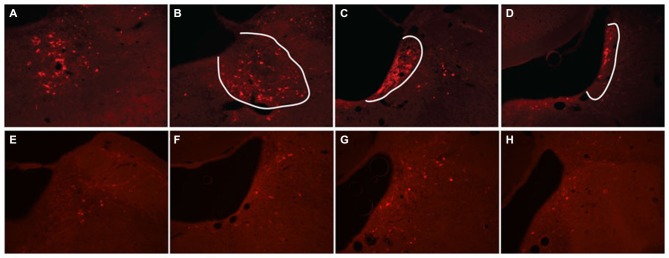
**hM3Dq receptor expression following locus coeruleus (LC) coinjections of TH-Cre-AAV and hM3Dq-AAV. (A–D)** hM3Dq expression as visualized by DS-Red immunofluorescence, and **(E–H)** mCherry native fluorescence indicating hM3Dq receptor expression. The approximate area of the LC is outlined in white in **(A–D)** to illustrate expression primarily restricted to LC neurons.

**Figure 6:**
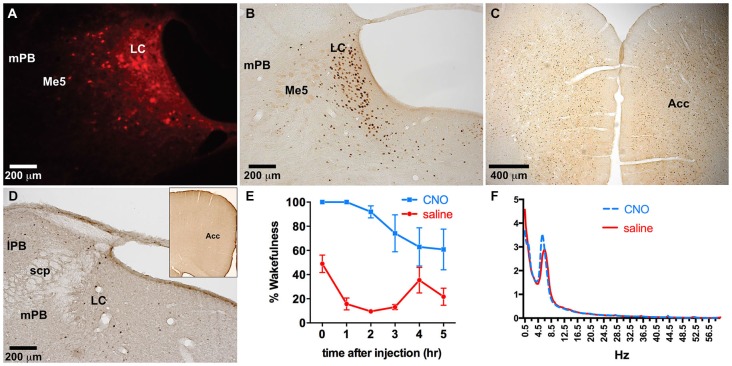
**Dual LC injection of TH-Cre-AAV and hM3Dq-AAV. (A)** hM3Dq expression is limited to LC neurons. **(B–D)** Following CNO injections, c-Fos expression markedly increased in the LC **(B)** as well as the entire neocortex, including the ACC **(C)**, which was not evident in LC or cortex of animals lacking hM3Dq receptors **(D). (E)** CNO induced wakefulness for most of the 6 h period following injection (blue line) compared to the normal amount of wakefulness observed following saline injection (red line). **(F)** Power spectral analysis revealed normal wake EEG signatures following CNO injection compared to saline (blue and red lines, respectively).

To examine the specificity of expression in another brain region rich in TH-positive neurons, as well as to verify the combinatorial viral vector strategy with another commonly used neuromodulation technique, we injected the LC with a combination of TH-Cre-AAV and hM3Dq-AAV. hM3Dq receptor expression was largely restricted to LC neurons (Figures 5A–H, 6A), although sparse hM3Dq+ cells were consistently observed in the medial and lateral parabrachial (PB) nucleus (evident in Figures 5B,C). Given that TH expression has been previously reported in the PB (Milner et al., 1986), it appears that the TH promoter used here is driving true eutopic expression of Cre in this brain region. On the basis of previous work (Gompf et al., 2010), we hypothesized that administration of the M3 ligand (CNO) would activate the TH+ neurons in the LC and produce sustained behavioral and electrographic arousal. Bolus agonist administration (CNO, ip, 0.3 mg/kg) at ZT 5 produced robust c-Fos expression in the LC, but not in adjacent areas of the pontine tegmentum (Figure 6B). A concomitant high level of c-Fos expression was observed throughout the cortex, including the ACC (Figure 6C). By contrast, very little c-Fos expression was evident in the LC or ACC following CNO injections in control rats (Figure 6D). A marked increase in EEG and behavioral wake was also observed over the 6-h post-injection recording period (81.6 ± 7.4% vs. 24.1 ± 1.4% waking following saline injections, *p* = 0.0001, *n* = 5; Figure 6E). The power spectra indicated a normal EEG following CNO injections. Though a slight elevation in power in the theta band (5–7 Hz) can be seen in this figure, it is not statistically significant (*p* = 0.09; Figure 6F).

### Combinatorial Viral Vectors Lacking Subtype Specificity

**Figure 7:**
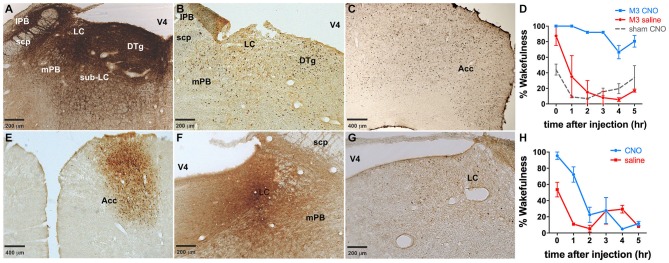
**Dual injections to either the LC or an LC projection. (A)** Dual injection of CMV-Cre-AAV and hM3Dq-AAV into the LC results in widespread expression of hM3Dq throughout the pons. **(B,C)** Widespread c-Fos expression throughout the pons **(B)** as well as the cortex (e.g., ACC, **(C)** was observed following CNO administration. **(D)** Wakefulness is increased in animals receiving this dual injection following CNO administration (blue line), whereas neither saline in these animals (red line) nor CNO injections in animals that did not receive viral injections (dashed gray line) produce prolonged wakefulness. **(E,F)** WGA-Cre-AAV injection into the ACC results in Cre expression in the ACC **(E)**, as well as transport of Cre to the LC **(F)**, where the AAV-hM3Dq vector was injected. **(G)** cFos expression is lower in these animals than in animals expressing hM3Dq throughout the pons **(B,C)**. Wakefulness **(H)** in these animals is greater following CNO injection (blue line) than saline (red line).

LC directed co-injections of viruses constitutively expressing Cre (CMV-Cre-AAV) and hM3Dq-AAV were performed to provide a comparison to TH-targeted hM3Dq. CMV-Cre and hM3Dq-AAV co-infusions resulted in expression of hM3Dq across vast portions of the pontine tegmentum, including most of the lateral and medial PB nucleus (lPB, mPB, Figure 7A), LC and the pre- and sub-coeruleus (Sub-LC, Figure 7A). CNO (0.3 mg/kg) administration elicited a large waking response (ZT 5–11: 88.5 ± 5.3% vs. 28.0 ± 12.6% following saline injections, *p* = 0.001, *n* = 3, Figure 7D) and concomitant widespread c-Fos expression across the pons and cortex (Figures 7B,C). CNO had little to no effect on animals not injected with viral vectors (Figure 7D). There was a decrease in the wakefulness immediately following the CNO infusion in sham virus injected animals, however this is likely an artifact, as we and several other groups have not observed significant changes in behavior after CNO treatment across multiple studies. Neither the behavioral responses nor the c-Fos expression could be attributed to a single brain region or cell type due to the widespread pontine hM3Dq expression. We also attempted to limit expression to a particular LC projection using a previously described method of retrograde transport of Cre from target regions (Gradinaru et al., 2010). Infusion of WGA-Cre-AAV into the ACC and hM3Dq-AAV into the LC resulted in robust Cre expression in both the ACC (Figure 7E) and the LC (Figure 7F), indicating successful WGA-mediated Cre transport. Following CNO injection, rats maintained 86.4 ± 8.7% and 61.8 ± 12.3% wakefulness for the first 2 and 4 h, respectively compared to 22.4 ± 5.8% following saline (Figure 7H), and a modest c-Fos expression was observed in the LC following CNO injections (Figure 7G). The smaller effect on wakefulness produced by CNO in this experiment is proportional to activation of only a subset of LC neurons projecting to the ACC (Chandler et al., 2014). C-Fos was not quantified in our study.

## Discussion

Using our dual combinatorial viral vector-based system we demonstrate, first, robust expression of two different transgenes, ChR2 and, hM3Dq in two different TH+ neuronal populations, the rat VTA and LC, respectively. Secondly, we demonstrate the ability to robustly alter neurochemical and behavioral outcomes based on these subtype specific alterations. Namely, we optically induced dopamine release in the nucleus accumbens, without any effect on dopamine release in the dorsal striatum. In addition, we observed a sustained increase in waking following chemogenetic activation of LC neurons.

Dual AAV targeting of ChR2 to TH+ neurons in the VTA produced an expression pattern consistent with TH+ neurons in the midbrain, as subsequent optical stimulation of the VTA resulted in the release of dopamine in the nucleus accumbens, but not in the dorsal striatum. This is consistent with the known innervation pattern of the striatum by midbrain dopaminergic neurons, in which the VTA projects primarily to the ventral portion of the striatum, while the substantia nigra projects primarily to the dorsal striatum. These neurochemical data strongly suggest that dual AAV targeting using TH-Cre can restrict expression to the VTA dopaminergic neurons in wildtype rats.

The observation that TH-Cre activated hM3Dq expression in the LC caused CNO-mediated wakefulness of such a long duration (6 h) is indicative of strong phasic activation of LC neurons. The LC has two different types of firing patterns: constant, ~3 Hz “phasic” activity and intermittent, ~10 Hz “tonic” impulse activity (Aston-Jones and Bloom, 1981), that have been correlated to different behavioral states (Aston-Jones and Cohen, 2005). Optogenetics has shown that phasic activation of LC neurons is capable of maintaining wakefulness in mice for up to 5 h whereas tonic LC activation promotes wakefulness for no more than an hour (Carter et al., 2010). Our results would therefore be consistent with CNO-driven excitation of TH+ neurons in the LC eliciting phasic firing patterns. Further studies should address whether hM3Dq receptors activate LC neurons phasically or tonically, and establish how sustained vs. acute activation (e.g., chemogenetic vs. optogenetic) promotes wakefulness.

We have also previously demonstrated in the rat that reciprocal innervation between the ACC and the LC provides the necessary circuitry to maintain wakefulness under novel environmental stimuli (Gompf et al., 2010). We therefore exploited these previous findings to demonstrate efficacy of a retrograde transport system for achieving projection-specific activation of LC neurons. We found that WGA-Cre transport from the ACC promoted expression of hM3Dq in ipsilateral LC neurons, although the number of hM3Dq-expressing neurons was far fewer (~60% less) than in those animals where AAV-TH-Cre was co-injected with hM3Dq-AAV into the LC, possibly because not all LC neurons project to the ACC (Chandler et al., 2014). We would note that while WGA-mediated Cre transport (Gradinaru et al., 2010) was chosen in this study to target projection-specific neurons, similar strategies have been employed using the retrograde transducing Canine Adenovirus 2 expressing Cre (Gore et al., 2013; Boender et al., 2014). Though both of these approaches are important in dissecting specific circuits, they are likely insufficient to target all neurons of a similar phenotype within a region.

To date, the use of Cre viruses, including lentiviral vectors (Tolu et al., 2010), have primarily occurred in conjunction with transgenic mice (Lazarus et al., 2007; Bass et al., 2013; Anaclet et al., 2014; Fenno et al., 2014), leaving a gap in our ability to generalize models across multiple species and limiting many scientists from conducting research using the most appropriate or translational species. For example, primates are enormously important in studies of behavior and human disease etiology, but we cannot easily manipulate their genetics. Currently a great deal of effort and money is being spent to generate transgenic rats and primates, despite relatively limited success. One notable exception is the recently generated TH-Cre rat (Brown et al., 2013), which is widely available. While these endeavors are worthwhile, the technical constraints of transgenic approaches put them out of reach for many researchers. Our approach bridges a gap in the ability to generalize models across multiple species by providing a relatively flexible system to generate targeted genetic manipulations to specific brain regions, pathways, and neuronal subtypes without having to produce and maintain transgenic animals. Genetic manipulations can be incorporated into existing wildtype models avoiding breeding costs and issues such as strain variations and developmental compensation. This flexibility may be advantageous as newer generation constructs are produced, such as the recently described INTRSECT system that uses multiple recombinases (e.g., Cre and Dre) in combination with expression vectors containing “intronic recombinase sites enabling combinatorial targeting” (Fenno et al., 2014). For instance, recent evidence suggests that in transgenic TH-Cre mice effector gene expression is not limited to dopaminergic neurons (Lammel et al., 2015; Stuber et al., 2015). If similar observations are found using the TH-Cre approach described here, multiple combinatorial viral vectors could potentially be used to limit expression specifically to dopaminergic VTA neurons. Additionally, AAV dual vector targeting should permit neuronal subtype specific restriction in primates, thus greatly increasing the number of potential therapeutic targets in humans and allowing for novel non-human primate models of human disease.

Our dual AAV targeting approach may have additional benefits over a single virus using a subtype specific promoter. First, there are limited minimal promoters available that can sufficiently drive subtype specific expression from a single virus, such as the PRSx8 promoter, which has been used to limit DREADD expression to noradrenergic neurons in the LC (Vazey and Aston-Jones, 2014). In addition, it is possible that a single virus will have unintended effects due to regulatory elements in the subtype specific promoter. For example, TH is known to express in a circadian manner (Sleipness et al., 2007; Sidor et al., 2015), and thus expression driven directly from the TH promoter may result in variations in transgene expression with time of day, depending on whether the minimal promoter still retains circadian controlled regulatory elements. Such regulatory influences would be avoided by our combinatorial system where transgene levels are determined by a generalized promoter.

While we have initially focused on establishing a toolbox for basic research experiments, our combinatorial AAV dual vector approach also has the potential to transform gene therapy application in the CNS. For years scientists have been using viral vectors to manipulate the genetics of neurons in primates, including humans, but have generally not pursued subtype specificity, rather choosing to limit the transgene to a specific brain region by controlling the infusion location. In theory, our approach should allow for true subtype specific targeting in primates. We are currently creating targeted non-human primate models of human diseases using AAV dual vector targeting. These models will allow us to test the translational potential of targeted gene delivery for more highly targeted gene therapy applications in humans, while also providing new non-human primate models of human disease for use in biological and pharmacological intervention studies.

The most pronounced limitation of the current iteration of dual AAV targeting is the lack of verified promoters that are active in wildtype species. In our study we used a previously verified rat TH promoter (Oh et al., 2009), whereas the dopamine beta-hydroxylase (PRSx8) promoter appears to also be successful in targeting noradrenergic neurons (Bruinstroop et al., 2012; Vazey and Aston-Jones, 2014). Going forward, and to improve the flexibility of the system, we will need to expand the toolbox by creating a multitude of subtype specific Cre viruses. To reach full utility, the relative specificity of each targeting Cre virus will need to be assessed individually in different brain regions and across multiple species. It should be noted that there are likely multiple factors that influence viral mediated subtype specificity including the size/composition of the promoter, the AAV serotype, virus titer, purification method and brain region injected. For example, while a VGLUT2 promoter may be able to target glutamatergic neurons in most brain regions, potentially it would also restrict expression to a subset of DAergic neurons in the VTA that also express VGLUT2 and co-release glutamate. Ultimately a library of subtype specific Cre viruses could be created, fully characterized in specific brain regions/species, and made available to the research community.

## Conflict of Interest Statement

The authors declare that the research was conducted in the absence of any commercial or financial relationships that could be construed as a potential conflict of interest.

## Abbreviations

AAV: Adeno-associated virus
ACC: Anterior cingulate cortex
AP: Anterior-posterior
ChR2: Channelrhodopsin
CMV: Cytomegalovirus
CNO: Clozapine N-oxide
DA: Dopamine
DIO: Double inverted open reading frame
DREADD: Designer receptor exclusively activated by designer drugs
DV: Dorsal-ventral
EEG: Electroencephalography
EMG: Electromyography
FSCV: Fast scan cyclic voltammetry
hM3Dq: DREADD derived from the human muscarinic 3 receptor, activating Gq
hSyn: Human synapsin
LC: Locus coeruleus
ML: Medial-lateral
NREM: Non-rapid eye movement sleep
REM: Rapid eye movement sleep
TH: Tyrosine Hydroxylase
VTA: Ventral tegmental area
WGA: Wheat germ agglutinin.
